# *Aspergillus fumigatus* Trehalose-Regulatory Subunit Homolog Moonlights To Mediate Cell Wall Homeostasis through Modulation of Chitin Synthase Activity

**DOI:** 10.1128/mBio.00056-17

**Published:** 2017-04-25

**Authors:** Arsa Thammahong, Alayna K. Caffrey-Card, Sourabh Dhingra, Joshua J. Obar, Robert A. Cramer

**Affiliations:** Department of Microbiology and Immunology, Geisel School of Medicine at Dartmouth, Hanover, New Hampshire, USA; Washington University School of Medicine

**Keywords:** *Aspergillus fumigatus*, cell wall, chitin, filamentous fungi, pathogenesis, trehalose

## Abstract

Trehalose biosynthesis is found in fungi but not humans. Proteins involved in trehalose biosynthesis are essential for fungal pathogen virulence in humans and plants through multiple mechanisms. Loss of canonical trehalose biosynthesis genes in the human pathogen *Aspergillus fumigatus* significantly alters cell wall structure and integrity, though the mechanistic link between these virulence-associated pathways remains enigmatic. Here we characterize genes, called *tslA* and *tslB*, which encode proteins that contain domains similar to those corresponding to trehalose-6-phosphate phosphatase but lack critical catalytic residues for phosphatase activity. Loss of *tslA* reduces trehalose content in both conidia and mycelia, impairs cell wall integrity, and significantly alters cell wall structure. To gain mechanistic insights into the role that TslA plays in cell wall homeostasis, immunoprecipitation assays coupled with liquid chromatography-tandem mass spectrometry (LC-MS/MS) were used to reveal a direct interaction between TslA and CsmA, a type V chitin synthase enzyme. TslA regulates not only chitin synthase activity but also CsmA sub-cellular localization. Loss of TslA impacts the immunopathogenesis of murine invasive pulmonary aspergillosis through altering cytokine production and immune cell recruitment. In conclusion, our data provide a novel model whereby proteins in the trehalose pathway play a direct role in fungal cell wall homeostasis and consequently impact fungus-host interactions.

## IMPORTANCE

Human fungal infections are increasing globally due to HIV infections and increased use of immunosuppressive therapies for many diseases. Therefore, new antifungal drugs with reduced side effects and increased efficacy are needed to improve treatment outcomes. Trehalose biosynthesis exists in pathogenic fungi and is absent in humans. Components of the trehalose biosynthesis pathway are important for the virulence of human-pathogenic fungi, including *Aspergillus fumigatus*. Consequently, it has been proposed that components of this pathway are potential targets for antifungal drug development. However, how trehalose biosynthesis influences the fungus-host interaction remains enigmatic. One phenotype associated with fungal trehalose biosynthesis mutants that remains enigmatic is cell wall perturbation. Here we discovered a novel moonlighting role for a regulatory-like subunit of the trehalose biosynthesis pathway in *A. fumigatus* that regulates cell wall homeostasis through modulation of chitin synthase localization and activity. As the cell wall is a current and promising therapeutic target for fungal infections, understanding the role of trehalose biosynthesis in cell wall homeostasis and virulence is expected to help define new therapeutic opportunities.

## INTRODUCTION

*Aspergillus fumigatus* is a filamentous fungus that can cause a severe fungal disease, invasive aspergillosis (IA), in immunocompromised humans (1, 2). Azoles are antifungal drugs that inhibit fungal ergosterol synthesis and are the current drugs of choice for IA treatment. Drug-drug interactions, undesirable side effects, and a growing emergence of azole-resistant strains in certain parts of the world are challenges faced by clinicians employing azole therapy against IA (3, 4). Thus, there is a growing need for new antifungal drugs to combat life-threatening infections caused by *A. fumigatus* and associated species.

Trehalose biosynthesis is found in many organisms, e.g., insects, plants, invertebrates, and fungi, but not in humans. The canonical fungal trehalose biosynthesis pathway was defined in *Saccharomyces cerevisiae* (5, 6). The canonical pathway in *S. cerevisiae* consists of the following components: Tps1p (trehalose-6-phosphate synthase), Tps2p (trehalose-6-phosphate phosphatase), and two regulatory subunits, Tps3p and Tsl1p (5–11). These proteins form a complex to produce trehalose (5, 6). Genes encoding trehalose biosynthesis proteins are essential for virulence in the human-pathogenic yeasts *Candida albicans* (12) and *Cryptococcus neoformans* (13). Canonical fungal trehalose biosynthesis is also present in *A. fumigatus*. In *A. fumigatus*, *tps1* has at least two paralogs that are important for trehalose production, *tpsA* and *tpsB* (*tpsA*/*B*) (14), whereas Tps2 has one ortholog, named OrlA (15). While loss of *tpsA* and *tpsB* enhances the virulence of *A. fumigatus* as measured by murine mortality and immunopathogenesis, the loss of *orlA* significantly attenuates virulence (14, 15). A striking feature of both the *tpsA*/*B* and *orlA* genetic mutants and of yeast trehalose mutants is their altered cell wall integrity. However, the mechanism(s) through which trehalose biosynthesis proteins impact fungal cell wall homeostasis is undefined. Given the extensive interactions between trehalose biosynthesis and basic fungal carbon metabolism, both indirect and direct mechanisms are plausible, though not mutually exclusive, causative models.

In this study, characterization of the unstudied *A. fumigatus* trehalose regulatory subunits *tslA* and *tslB* revealed a surprising role for TslA in modulating fungal cell wall homeostasis. Our results support a model whereby TslA plays a critical direct role in fungal cell wall homeostasis through modulating the localization and activity of a class V chitin synthase enzyme, CsmA. Thus, for the first time, our results provide novel insights into mechanisms through which the canonical fungal trehalose biosynthesis pathway directly impacts fungal cell wall homeostasis and consequently the host-pathogen interaction.

## RESULTS

### TslA and TslB are homologs of yeast trehalose regulatory subunits Tsl1 and Tps3.

To identify putative regulatory subunits of the trehalose complex in *A. fumigatus*, we queried the protein sequences of *S. cerevisiae* Tsl1p and Tps3p against the *A. fumigatus* strain A1163 protein database using BLASTp algorithms (http://www.aspergillusgenome.org/). Two proteins, AFUB_089470 and AFUB_021090, showed significant sequence similarity to Tsl1p and Tps3p and were consequently named TslA and TslB, respectively. TslA contains 919 amino acids, while TslB contains 918 amino acids. The TslA and Tsl1p and Tps3p protein sequences showed 40% and 37% amino acid identity and 59% and 54% protein sequence similarity, respectively. TslB and Tsl1p and Tps3p showed 38% and 36% amino acid identity and 57% and 53% protein sequence similarity, respectively. Protein domain analyses revealed that TslA and TslB share domains similar to those of the trehalose-6-phosphate phosphatase (TPP) OrlA, such as the glycosyl transferase domain (GT1-TPS) and the halogen-associated dehydrogenase-like domain (HAD-TPP), as previously reported in *A. niger* (16). However, compared to the known catalytic sites of TPS and TPP domains in bacteria (17, 18), TslA and TslB appear to lack catalytic residues of both domains similar to those of yeast Tsl1p and Tps3p. To study the function of these proteins in *A. fumigatus*, we generated genetic single- and double-null mutants of *tslA* and *tslB* in *A. fumigatus* CEA17 (mutants Δ*tslA*, Δ*tslB*, and Δ*tslA*/*B*) as previously described (19–21). Reconstituted Δ*tslA* and Δ*tslB* strains were generated by ectopic insertion of the wild-type *tslA* and *tslB* alleles (*ΔtslA+tslA* and Δ*tslB+tslB*) (22). Singly reconstituted Δ*tslA*/*B* strains were also generated using either wild-type *tslA* alleles or wild-type *tslB* alleles (Δ*tslA*/*B+tslA* or Δ*tslA*/*B+tslB*) (22). All strains were confirmed by both PCR and Southern blot analyses. Furthermore, the confirmed strains were analyzed with quantitative reverse transcriptase PCR (qRT-PCR) and mRNA corresponding to *tslA* and *tslB* was confirmed to be absent in all mutants and confirmed to be restored to wild-type levels in the respective reconstituted strains (data not shown). In Δ*tslA*, we observed increased mRNA levels *of tslB*; *tslA* mRNA levels remained similar to the wild-type levels in the Δ*tslB* mutant (data not shown).

### Loss of TslA and TslB decreases trehalose content and delays germination.

**FIG 1:**
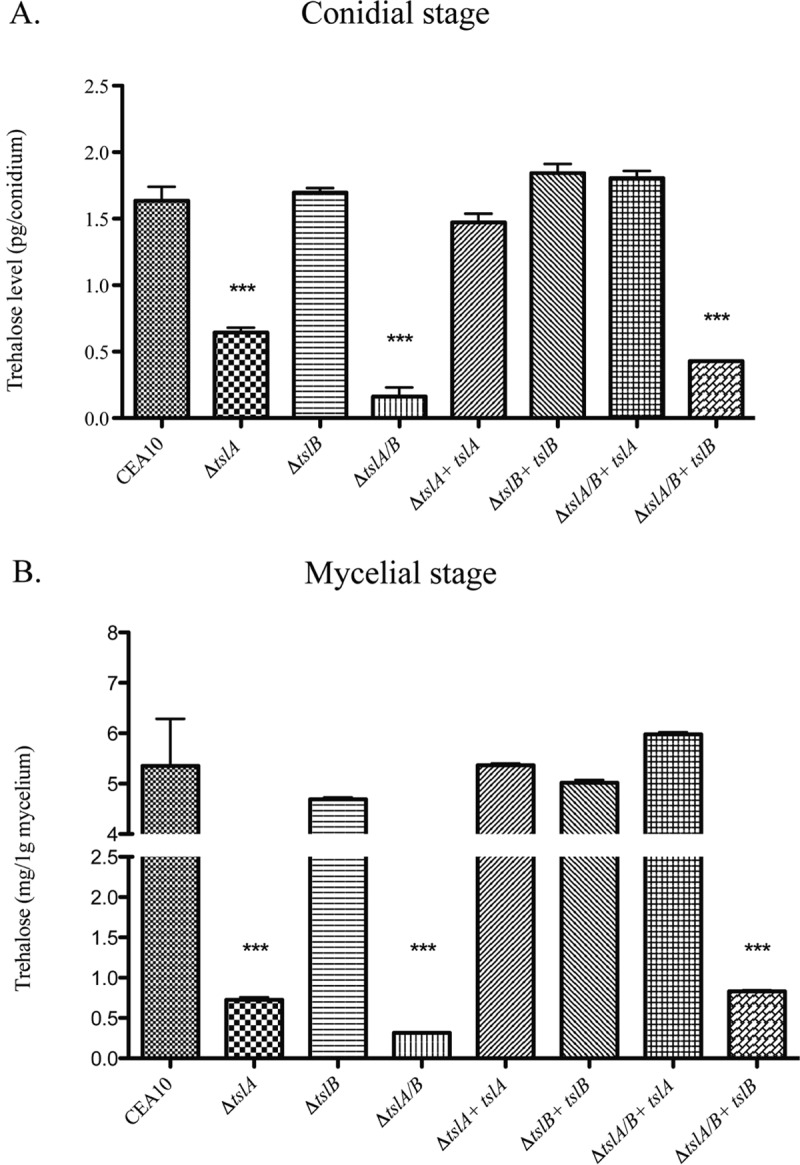
Loss of TslA and TslB decreases trehalose production in both conidia (A) and hyphae (B). Quantitation of trehalose production in conidia and mycelia was performed using glucose oxidase (GO) assays (Sigma) after trehalase enzyme incubation. For the conidial stage, 2 × 10^8^ conidia were used to extract trehalose by boiling at 100°C for 20 min and collecting the supernatant to perform GO assays. For the mycelial stage, 1 × 10^8^ conidia were cultured in 10 ml LGMM at 37°C for 16 h, the mycelia were weighed and lyophilized, and trehalose extraction was performed. Data are presented as means ± SE of results from three biological replicates. ***, *P* < 0.0001 (unpaired two-tailed Student’s *t* test compared to the wild-type CEA10 results).

To test the hypothesis that TslA and TslB are involved in trehalose biosynthesis in *A. fumigatus*, we measured conidia and mycelium trehalose content in our wild-type and generated strains (Fig. 1). A significant decrease in trehalose content in the Δ*tslA*, Δ*tslA*/*B*, and Δ*tslA*/*B+tslB* strains was observed compared to levels observed with the wild-type and reconstituted strains in both the conidial and mycelial stages (Fig. 1). Loss of TslB alone had minimal impact on trehalose levels in conidia or mycelia. These results suggest that TslA is more critical for trehalose production than TslB. However, loss of TslB in Δ*tslA* further reduced trehalose content compared to that seen with Δ*tslA* alone (*P* < 0.0001). This result suggests that TslB is also involved in trehalose production (Fig. 1).

Loss of trehalose biosynthesis in *A. fumigatus* and other filamentous fungi affects germination of conidia (14, 15, 23). Consistent with previous observations, the Δ*tslA*, Δ*tslB*, and Δ*tslA*/*B* strains showed a significant delay in germination in the first 8 h when cultured in liquid glucose minimal medium (LGMM). At 8 h, the wild-type strain germinated at 94.00 ± 2.00% whereas the Δ*tslA*, Δ*tslB*, and Δ*tslA*/*B* strains germinated at 80.33 ± 2.08% (*P* = 0.0012), 86.00 ± 3.46% (*P* = 0.0257), and 86.67 ± 2.31% (*P* = 0.0142), respectively. Nevertheless, these mutants showed no detectable differences in radial growth on solid GMM or in biomass in batch culture at 37°C compared to the levels seen with the wild-type and reconstituted strains.

### Loss of TslA increases susceptibility to cell wall-perturbing agents.

**FIG 2:**
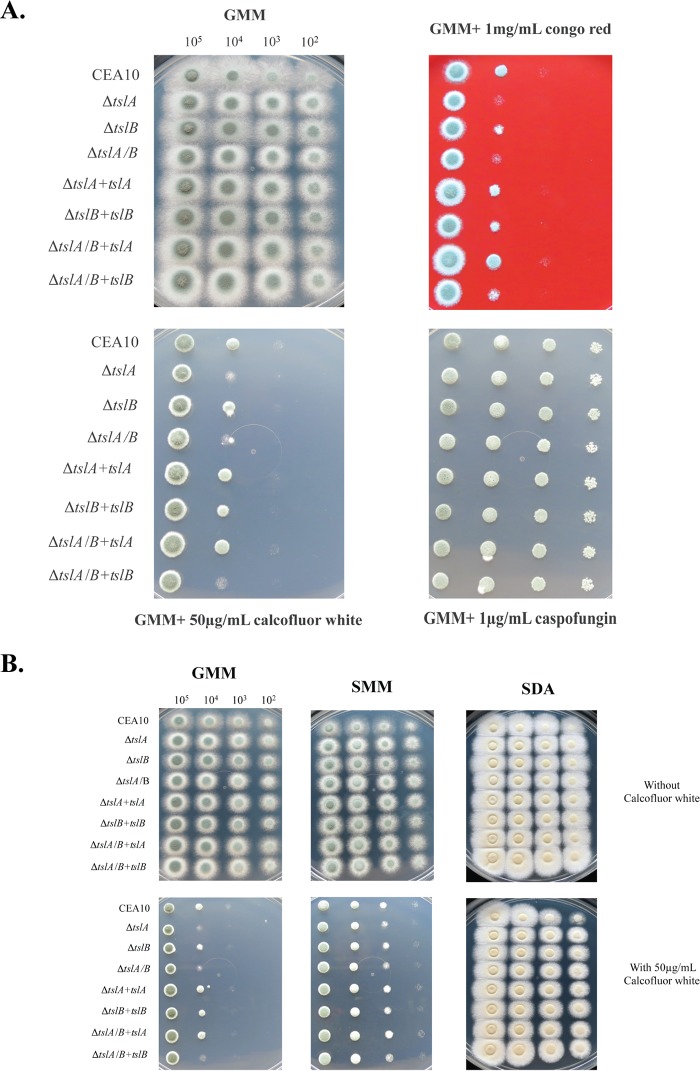
Loss of TslA increases fungal susceptibility to cell wall-perturbing agents (A), and growth of the Δ*tslA* strain is restored on sorbitol minimal media (SMM) and Sabouraud dextrose media (SDA) in the presence of 50 μg/ml calcofluor white (B). Dropout assays were performed at 37°C for 2 days by using 10^5^ to 10^2^ conidia for each strain inoculated on GMM with or without cell wall-perturbing agents, i.e., 1 mg/ml Congo red, 50 μg/ml calcofluor white, and 1 μg/ml caspofungin. Images and data are representative of three independent experiments with similar results.

Trehalose biosynthesis null mutants have associated cell wall defects in *A. fumigatus* as evidenced by data from Δ*tpsA*/*B* and Δ*orlA* strains (14, 15). To test the hypothesis that TslA and TslB play a role in cell wall homeostasis, we utilized the cell wall-perturbing agents Congo red (CR), calcofluor white (CFW), and caspofungin (CPG). We observed increased CR and CFW susceptibility with the Δ*tslA* and Δ*tslA*/*B* strains (Fig. 2A). No significant difference in CPG susceptibility was observed. To further confirm the cell wall phenotypes of these mutants, we utilized osmostabilizing medium containing 1.2 M sorbitol (sorbitol minimal media [SMM]) and an enriched medium, Sabouraud dextrose agar (SDA). Δ*tslA* showed restored cell wall phenotypes on both SMM and SDA in the presence of CFW (Fig. 2B). As both CR and CFW bind to chitin on the cell wall and inhibit growth, while CPG inhibits β-1,3-glucan synthase, these results suggest that loss of TslA affects the chitin component of the fungal cell wall.

### Loss of TslA alters cell wall structure and exposure of fungal cell wall microbe-associated molecular patterns (MAMPs).

**FIG 3:**
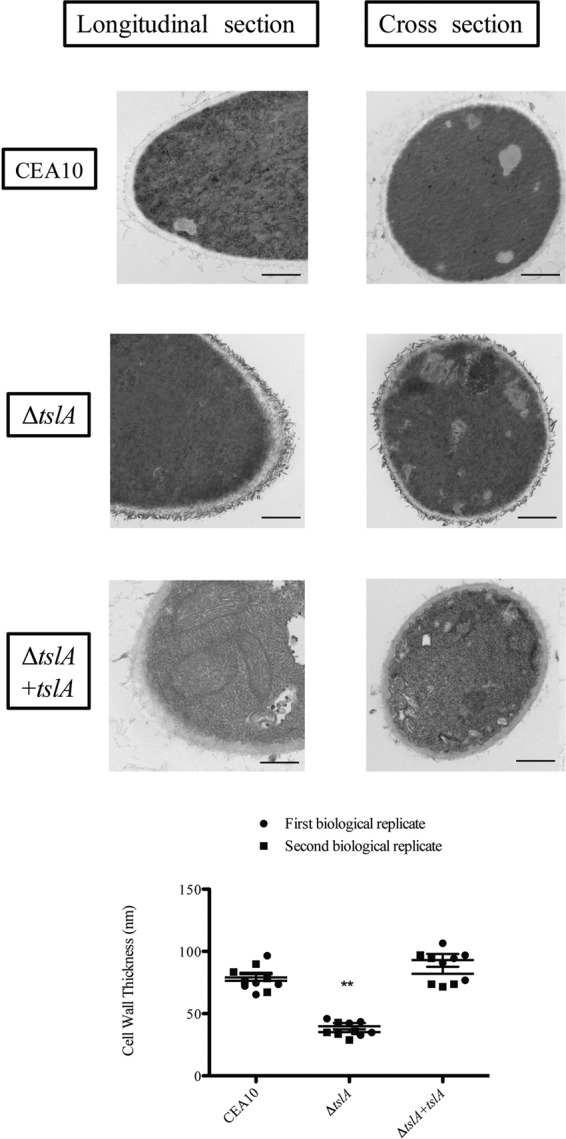
Loss of TslA decreases fungal cell wall thickness and results in accumulation of electron-dense material at the outer layer of the cell wall. Mycelia from each strain were prepared for TEM as previously described (19, 39). Cell wall thickness was analyzed by ImageJ. Data are presented as means ± SE of 10 measurements from two biological replicates of each strain. **, *P* value = 0.002 (unpaired two-tailed Student’s *t* test compared to the wild-type CEA10 results). Bars, 500 nm.

One possible mechanism to explain the increased susceptibility of the Δ*tslA* strain to cell wall-perturbing agents is an inherent alteration in cell wall structure. To explore this hypothesis, transmission electron microscopy (TEM) was utilized. TEM micrographs revealed that the Δ*tslA* strain had a significantly thinner cell wall than the wild type (*P* = 0.002) (Fig. 3). Moreover, an accumulation of an electron-dense material near the cell wall of the Δ*tslA* strain was observed along the hyphae. We hypothesize that loss of TslA may alter extracellular matrix and/or associated cell wall proteins of this fungus. Given the significant alteration in the Δ*tslA* strain cell wall structure, we next tested the hypothesis that exposure of key MAMPs, chitin and β-glucan, is altered in this mutant.

**FIG 4:**
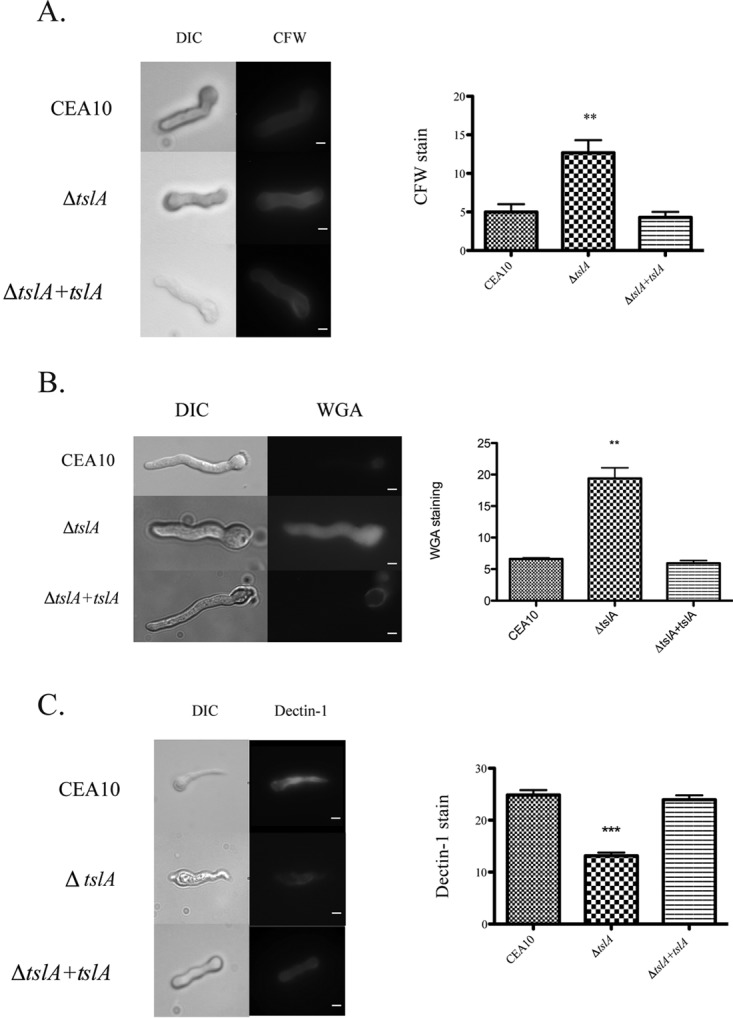
Loss of TslA alters MAMP cell wall exposure. (A and B) The *ΔtslA* strain has increased chitin levels/exposure as measured by calcofluor white (CFW) staining (A) or wheat germ agglutinin (WGA) (B) compared to the results seen with wild-type CEA10 and the reconstituted *ΔtslA+tslA* strain. Each strain was cultured into the germling stage under normoxic conditions at 37°C. The germlings were UV irradiated and stained with 25 μg/ml CFW or with 5 μg/ml WGA. The mean intensity was analyzed using ImageJ and the corrected total cell fluorescence (CTCF) was calculated (69, 70). **, *P* value = 0.0074 for CFW and 0.0017 for WGA compared to CEA10 (unpaired two-tailed Student’s *t* test compared to the wild-type CEA10 results). DIC, differential interference contrast. (B) The *ΔtslA* strain has decreased β-glucan exposure as measured by s-dectin-1 staining compared to the wild-type CEA10 and the reconstituted *ΔtslA+tslA* strain. Each strain was cultured to the germling stage under normoxic conditions at 37°C. The germlings were UV irradiated, blocked, and stained with a conditioned medium containing s-dectin1-hFc followed by Alexa Fluor 488-conjugated, goat anti-human IgG1. The corrected total cell fluorescence (CTCF) was calculated. ***, *P* value = 0.0005 (unpaired two-tailed Student’s *t* test compared to the wild-type CEA10 results). Data are presented as means ± SE of 15 images from three biological replicates. Bar, 3 μm.

CFW and wheat germ agglutinin (WGA) staining were used to observe chitin levels and exposure on the cell wall. Loss of TslA dramatically increased both CFW staining and WGA staining, which likely reflects increased chitin content of this mutant (*P* = 0.0074 for CFW and *P* = 0.0017 for WGA) (Fig. 4A and B). Soluble dectin-1 (s-dectin-1) staining was used to observe β-glucan exposure, and loss of TslA significantly decreased s-dectin-1 staining on fungal germlings (*P* = 0.0005) (Fig. 4C). We conclude that loss of TslA affects cell wall homeostasis in part by disrupting chitin and β-glucan homeostasis.

**FIG 5:**
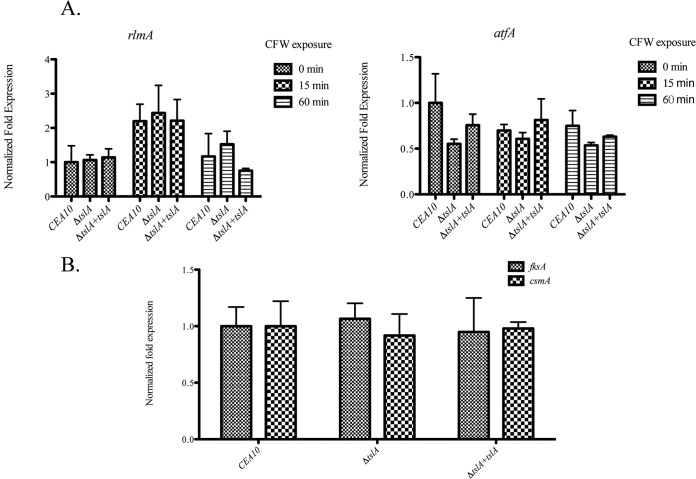
Loss of TslA does not affect the cell wall integrity and HOG-MAPK pathways (A) and does not change the expression of *fksA* and *csmA* (B). A total of 10^6^ conidia of the wild-type strain and the Δ*tslA* strain were incubated overnight in liquid GMM, and CFW was added for 0, 15, and 60 min as indicated. Samples were collected, and RNA extraction was performed for measuring *rlmA*, *atfA*, *fksA*, and *csmA* mRNA abundance using qRT-PCR analysis as previously described (15). Data are presented as means ± SD of results from three biological replicates of each strain.

One possible explanation of these results is that loss of TslA indirectly affects the fungal cell wall through induction of a cell wall integrity response that is perhaps due to an alteration in intracellular osmotic homeostasis resulting from reductions in trehalose levels. To test this hypothesis, we utilized qRT-PCR to quantitate mRNA levels of transcription factors known to be induced by cell wall stress, *rlmA* and *atfA* (24, 25). We observed that the mRNA levels of both *rlmA* and *atfA* in the Δ*tslA* strain were equivalent to the levels seen with the wild type and the reconstituted strains with or without the presence of CFW (Fig. 5A). Alternatively, it is possible that loss of TslA alters carbon metabolic flux and thus affects cell wall biosynthesis. Several studies in multiple fungi have observed significant changes in cell wall biosynthesis-encoding gene mRNA levels in response to nutrient availability (26–29). To investigate this hypothesis, we analyzed the mRNA levels of *fksA*, encoding a β-glucan synthase enzyme, and of *csmA*, encoding a class V chitin synthase enzyme, using qRT-PCR. We observed no change in the expression levels of these genes in the Δ*tslA* strain (Fig. 5B). The combination of osmostabilizing medium rescue of the cell wall perturbation phenotype, lack of an intrinsic cell wall integrity response, and lack of changes in cell wall biosynthesis-encoding gene mRNA levels suggests that changes in the cell wall homeostasis resulting from the loss of TslA are unlikely to be solely the result of altered carbon metabolism.

### TslA interacts with a class V chitin synthase enzyme.

To understand the mechanism behind the role of TslA in cell wall homeostasis, we utilized an affinity purification approach to identify proteins interacting with TslA. To utilize this approach, we generated a TslA C-terminal S-tag strain (30, 31). Similar to previous observations in *S. cerevisiae*, we observed that TslA interacts with the TPP, OrlA, which suggests that TslA may regulate TPP activity/function in *A. fumigatus* (see Table S2 in the supplemental material). TslA also interacted with metabolic enzymes involved in central carbon metabolism, including proteins in glycolysis and pentose phosphate pathways. Unexpectedly, TslA interacted with the chitin synthase enzyme, CsmA (ChsE; AFUB_029080). These data suggest that TslA has important metabolism regulatory functions in addition to the canonical function in trehalose biosynthesis. A list of TslA interacting proteins, with score and identity notations, is presented in Table S2.

**FIG 6:**
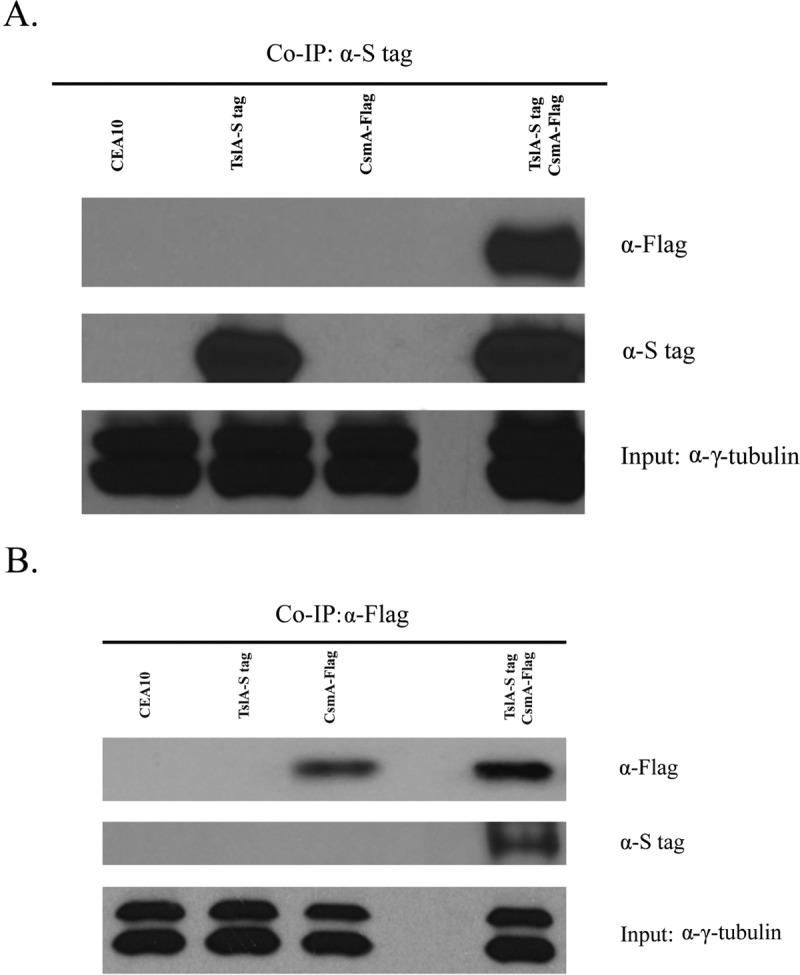
TslA physically interacts with CsmA. Affinity purification assays from Flag-tagged CsmA strains in the background of S-tagged TslA were performed with S-protein beads (A) and anti-Flag beads (B) to verify interactions. Data are images representative of results from three independent experiments, all with similar results.

To validate the protein-protein interaction between TslA and CsmA, we utilized a coimmunoprecipitation (Co-IP) approach (Fig. 6). We first introduced a 3× Flag tag to the C terminus of CsmA in the background of the wild-type and S-tagged TslA strains. We observed no changes in the phenotypes and trehalose levels of these tagged strains compared to wild-type levels (data not shown). We performed coimmunoprecipitation assays using S-protein beads with the wild-type, S-tagged TslA, and Flag-tagged CsmA strains and the S-tagged TslA and Flag-tagged CsmA strain. Using Western blot analysis, we observed that TslA coimmunoprecipitated from only the S-tagged TslA strain and the S-tagged TslA and Flag-tagged CsmA strain. In support of the affinity purification data, CsmA coimmunoprecipitated in the S-tagged TslA and Flag-tagged CsmA strain (Fig. 6A). To further confirm the interaction between TslA and CsmA, we performed reciprocal coimmunoprecipitation assays using anti-Flag M2 magnetic beads. We observed CsmA to coimmunoprecipitate from only the Flag-tagged CsmA strain and the S-tagged TslA and Flag-tagged CsmA strains. Also, TslA was coimmunoprecipitated from the S-tagged TslA and Flag-tagged CsmA strains (Fig. 6B). From these results, we conclude that TslA and CsmA physically interact in *A. fumigatus*.

### Loss of TslA increases chitin synthase activity and affects cellular localization of CsmA.

**FIG 7:**
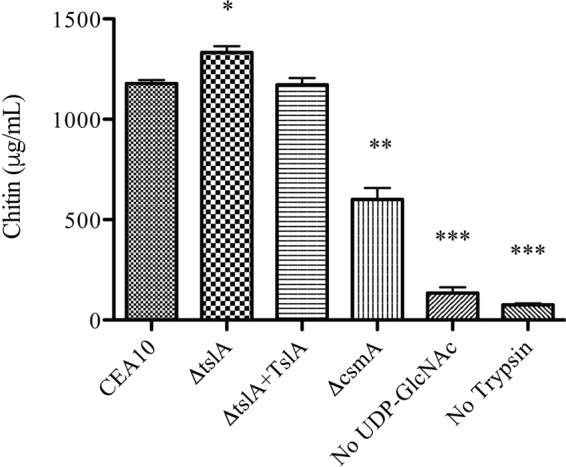
Loss of TslA increases chitin synthase activity. Ten micrograms of membrane proteins were used to perform a nonradioactive chitin synthase activity assay. Each strain was cultured at 30°C for 6 h and switched to 37°C for 24 h. Ten micrograms of the wild type’s membrane proteins was used to compare with no substrate, UDP-N-acetyl glucosamine (UDP-GlcNAc) and no trypsin as negative-control assays. *, *P* = 0.0117; **, *P* = 0.0013; ***, *P* < 0.0001 (unpaired two-tailed Student’s *t* test compared to the wild-type CEA10 results). Data are presented as means ± SE of results from three biological replicates.

One potential mechanism to explain our results is that TslA directly regulates chitin synthase activity through CsmA. To test the hypothesis that TslA regulates CsmA activity, we utilized a nonradioactive chitin synthase activity approach successfully utilized in *A. fumigatus* (32, 33). After extracting membrane proteins and incubating with substrates for chitin production, we observed a significant increase in chitin production in the Δ*tslA* strain compared to the wild-type, reconstituted, and control Δ*csmA* strains whereas the negative controls showed very low chitin content (Fig. 7) (for 10 μg, *P* = 0.0117 [for comparisons between the wild-type strain and the Δ*tslA* strain) and *P* = 0.0013 (for comparisons between the wild-type strain and the Δ*csmA* strain). This result supports the hypothesis that TslA is a potential negative regulator of chitin synthase activity through its interaction with CsmA.

**FIG 8:**
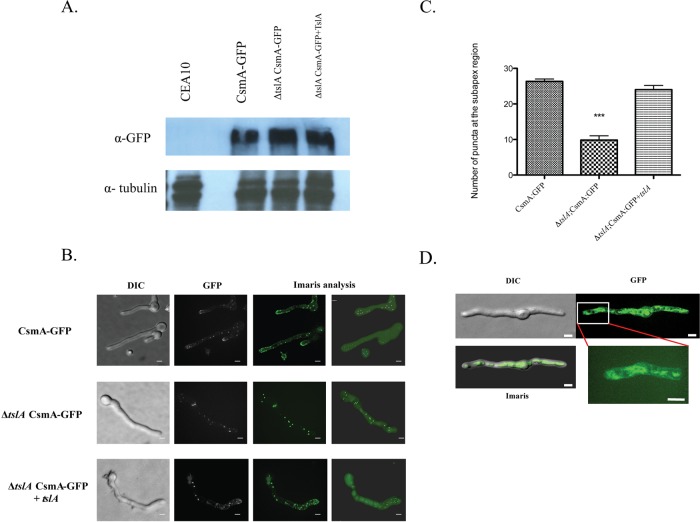
TslA promotes CsmA hyphal tip localization. (A) Western blot analysis of C-terminal GFP-tagged CsmA in the wild-type strain, Δ*tslA* strain, and *ΔtslA+tslA* strain backgrounds. (B) C-terminal GFP-tagged CsmA was generated in the wild-type strain, Δ*tslA*, and *ΔtslA+tslA* mutant backgrounds. Each strain was cultured at 37°C for 12 h, and live-cell imaging was performed using a Quorum Technologies WaveFX spinning disk confocal microscope (magnification, ×1,000). The images were analyzed using Imaris 8.1.4 software. (C) Loss of TslA changes the number of CsmA puncta at the hyphal tip. To quantify the puncta at the subapex region (within 20 μm of the tip), the puncta in the images were counted and analyzed using Imaris 8.1.4 software. ***, *P* < 0.0001 (unpaired two-tailed *t* test compared to the wild-type CEA10 results). (D) Localization of GFP-tagged TslA. C-terminal GFP-tagged TslA was generated and cultured at 37°C for 12 h. Live-cell imaging was performed using a Quorum Technologies WaveFX spinning disk confocal microscope (magnification, ×1,000). The three-dimensional (3D) structure of TslA puncta was created by the use of Imaris 8.1.4 software. Data are presented as means ± SE of results corresponding to 15 images from three biological replicates. Bar, 3 μm.

As chitin synthase localization is critical for cell wall homeostasis in fungi, we hypothesized that TslA alters CsmA localization and, consequently, chitin synthase activity. To observe the change in the localization of CsmA, we introduced a green fluorescent protein (GFP) tag into the C terminus of CsmA in the wild-type and Δ*tslA* strain backgrounds. We confirmed the stability of the C-terminal GFP-tagged CsmA protein of each strain using Western blot analysis (Fig. 8A). Consistent with results in *A. nidulans*, CsmA primarily localized to the growing hyphal tips and septa in wild-type *A. fumigatus* (34). In contrast, the localizations of CsmA in the Δ*tslA* strain were dispersed along the lateral cell wall of the fungus and throughout the cytoplasm and were not spatially restricted to the hyphal tips or septa (Fig. 8B). Furthermore, to quantify the puncta at the subapex region (within 20 μm of the tip), the puncta in the images were analyzed, and fewer puncta were visible in the Δ*tslA* strain (*P* < 0.0001 [for comparisons between CEA10 and the Δ*tslA* strain]) (Fig. 8C). Consequently, we conclude that TslA is critical for proper CsmA localization at the hyphal tip and hypothesize that loss of TslA causes dysregulation of chitin synthase activity through altered CsmA localization. To gain further insight into how TslA affects CsmA localization, we investigated TslA localization using a strain with expression of C-terminal GFP-tagged TslA. We observed that TslA localized nonspecifically in the cytosol throughout the hyphae after 12 h or 16 h of incubation (Fig. 8D) (see Fig. S1 in the supplemental material).

10.1128/mBio.00056-17.1 FIG S1  TslA localizes nonspecifically through the hyphal cytosol. C-terminal GFP-tagged TslA was generated and cultured at 37°C for 12 h or 16 h. Live-cell imaging was performed using a Quorum Technologies WaveFX spinning disk confocal microscope (magnification, ×1,000). The images were analyzed using Imaris 8.1.4 software to form a 3D structure. Data are presented as means ± SE of results corresponding to 15 images from three biological replicates. Bar, 5 μm. Download FIG S1, TIF file, 2.4 MB. Copyright © 2017 Thammahong et al. 2017 Thammahong et al. This content is distributed under the terms of the Creative Commons Attribution 4.0 International license.

### TslA modulates the host inflammatory response.

**FIG 9:**
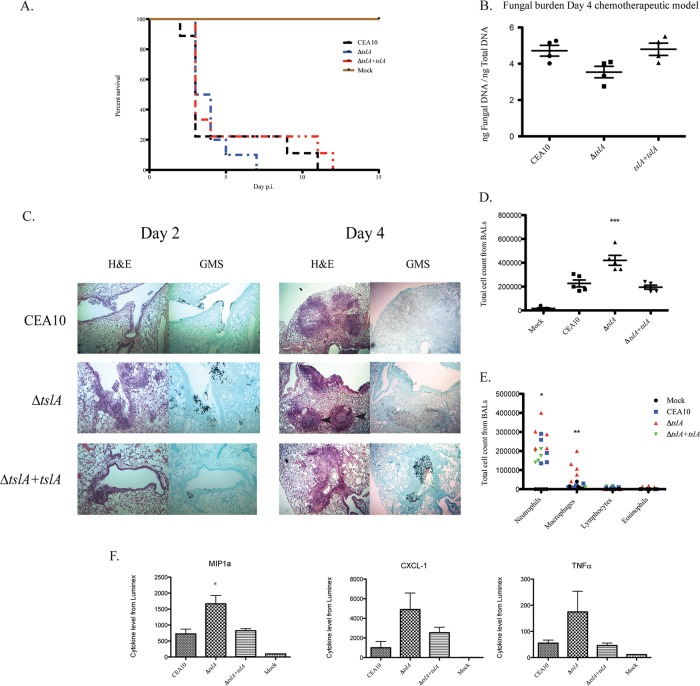
Survival analysis (A) and fungal burden (B) data from the Δ*tslA* strain are similar to the data from the wild-type and *ΔtslA+tslA* strains, while the loss of TslA increased inflammation (panels C and D and panels E and F). (A) A total of 10^6^ conidia of each strain were inoculated via the intranasal route in a chemotherapeutic IPA murine model. Ten CD1 mice were used in each group. Survival analysis was performed for 2 weeks. p.i., postinfection. (B) No significant differences in fungal burden were observed in the strains tested. Analysis of the fungal burden of these mice was performed as previously described (36). (C) Δ*tslA*-infected lungs show more inflammatory cell infiltrations. The fungal histology was performed on day 2 and day 4 to observe the inflammatory cell infiltrations. Arrowheads show the abscess-like structure. Images are representative of results from three mice. Magnification, ×50. (D and E) Δ*tslA*-infected bronchoalveolar lavage fluid samples (BALs) had increased cell infiltrations, especially macrophages. To observe changes in the inflammatory response *in vivo*, cell counts and differential counts were performed. (D) *P* value = 0.0051 (unpaired two-tailed *t* test compared to the wild-type CEA10 results). (E) For neutrophils, *, *P* < 0.05 (two-tailed ANOVA for comparisons among the CEA10, Δ*tslA*, and Δ*tslA+tslA* strains); for macrophages, **, *P* < 0.01 (two-tailed ANOVA for comparisons among the CEA10, Δ*tslA*, and Δ*tslA+tslA* strains). (F) Luminex assay results from Δ*tslA* mutant-infected BALs show an increased inflammatory cytokine profile. Data are presented as means ± SE of results from BALF from three mice of each strain. *, *P* = 0.0286 (unpaired two-tailed Mann-Whitney test).

As the fungal cell wall is at the interface of the host-pathogen interaction, we next tested the hypothesis that loss of TslA impacts murine invasive pulmonary aspergillosis (IPA) outcomes. First, fungal virulence was assessed using a survival analysis in the chemotherapeutic murine model of IPA (35). From the survival experiment, all Δ*tslA* strain-inoculated mice perished by day 7, whereas the wild-type-strain-inoculated and reconstituted-strain-inoculated groups survived through the second week (Fig. 9A). The median durations of survival for mice inoculated with the wild type, the Δ*tslA* strain, and the reconstituted strain were 3, 3.5, and 3 days, respectively. Although the Δ*tslA* strain-inoculated mice had a clear trend toward earlier mortality than the mice in the groups inoculated with the wild-type strain or the reconstituted strain, Kaplan-Meier analysis showed no significant difference between groups (*P* = 0.066 [for comparisons between the wild-type and Δ*tslA* strain groups]). We next examined the pulmonary fungal burden of lung homogenates using a quantitative PCR (qPCR) approach to quantitate fungal 18S ribosomal DNA (rDNA) levels (36) and observed no significant difference between the results seen with the Δ*tslA* strain and the wild-type and reconstituted strains (*P* = 0.057 [for comparisons between the wild-type and Δ*tslA* groups) (Fig. 9B). However, significant differences in lung histopathology were observed between groups, with Δ*tslA*-inoculated mice containing increased levels of inflammatory foci compared to those inoculated with the wild-type and reconstituted strains (Fig. 9C). Moreover, the organizations of the inflammatory lesions were significantly different between the Δ*tslA* strain and the wild type, with many Δ*tslA* lesions exhibiting abscess-like characteristics, especially on day 4 after inoculation (Fig. 9C). We hypothesize that the trend toward higher mortality rates and earlier mortality of Δ*tslA*-inoculated mice was the result of increased immunopathogenesis and an altered host response.

We tested this hypothesis by collecting bronchoalveolar lavage fluid (BALF) on day 2 postinoculation (D2PI). Consistent with the histopathological findings, we observed larger inflammatory cell infiltrates from BALF of Δ*tslA*-inoculated mice than from BALF of the wild-type- or reconstituted-strain-inoculated groups (*P* = 0.0051 [for comparisons between CEA10 and the Δ*tslA* strain]) (Fig. 9D). Cell differential counts revealed increased infiltration of macrophages and neutrophils from the BALF of Δ*tslA*-inoculated mice compared to the results seen with the wild-type and reconstituted strains (for neutrophils, *P* < 0.05 [two-way analysis of variance {ANOVA} for comparisons among the CEA10, Δ*tslA*, and *ΔtslA+tslA* strains; for macrophages, *P* < 0.01 [two-way ANOVA for comparisons among the CEA10, Δ*tslA*, and *ΔtslA+tslA* strains]) (Fig. 9E).

To better understand potential causes of the higher levels of inflammatory cellular infiltrate inside Δ*tslA*-inoculated lungs, we utilized a Luminex assay to quantitate selected inflammatory cytokines from the BALF. Despite the equivalent levels of fungal burden, we observed an increased-inflammatory-cytokine profile, including increases in the levels of tumor necrosis factor alpha (TNF-α), CXCL1, and macrophage inflammatory protein 1-alpha (MIP-1α or CCL3), in Δ*tslA*-inoculated BALF compared to the wild-type and reconstituted-strain results. MIP-1α levels increased significantly in the Δ*tslA*-inoculated BALF (*P* = 0.0286) (Fig. 9F). Consequently, we conclude that the increased chitin levels and the decreased β-glucan levels on the cell wall of the Δ*tslA* strain alter the immunopathogenesis of murine IPA through increased and differential recruitment of inflammatory cells, likely through alterations in the secretion of proinflammatory cytokines.

## DISCUSSION

The trehalose biosynthesis pathway is crucial for the virulence of human- and plant-pathogenic fungi, including *Candida albicans* (12), *Cryptococcus neoformans* (13), *Aspergillus fumigatus* (15), and *Magnaporthe oryzae* (37). In *A. fumigatus*, loss of the trehalose synthases TpsA and TpsB virtually eliminates trehalose production and results in a strain with an increase in virulence as measured by murine survival in a corticosteroid murine model of IPA (14). Though the mechanism for the increase in virulence is unknown, it is suggested to be driven by altered cell wall composition and immunopathogenesis (14). This result was rather surprising given that trehalose synthase null mutants in other human-pathogenic fungi are severely attenuated in virulence. In contrast, loss of the TPP Tps2 ortholog OrlA in *A. fumigatus* severely attenuated virulence in a chemotherapeutic murine model and markedly reduced virulence in an X-CGD murine model (15). As with the loss of TpsA and TpsB, the loss of OrlA significantly alters the cell wall of *A. fumigatus*. Yet it has remained enigmatic how trehalose biosynthesis and cell wall biosynthesis are mechanistically linked.

To further explore the role of trehalose biosynthesis in *A. fumigatus* virulence and cell wall homeostasis, we identified and characterized two additional homologs of the *S. cerevisiae* trehalose biosynthesis complex, here named TslA and TslB. Our major finding is that *A. fumigatus* TslA physically interacts with the chitin synthase CsmA, which leads to a novel model where TslA can moonlight as a regulator of chitin biosynthesis. While our data do not rule out perturbations in carbon metabolism that occur upon loss of trehalose biosynthesis proteins impacting cell wall biosynthesis, they strongly suggest that TslA has a direct regulatory role through its interaction with the CsmA chitin synthase.

In *A. fumigatus*, TslA and TslB lack the canonical catalytic residues of both TPS and TPP domains, similarly to *S. cerevisiae* Tps3p (*Sc*Tps3p) and Tsl1p. Yet loss of TslA in *A. fumigatus* leads to a significant decrease in the trehalose content in both conidia and mycelia, similarly to the loss of *Sc*Tsl1p (11). Consequently, TslA is directly involved in regulating trehalose biosynthesis in *A. fumigatus*. Intriguingly, we observed a direct interaction between TslA and the *A. fumigatus* TPP (OrlA) in our experiments. Further experiments are needed to test the hypothesis that TslA serves as a regulator of TPP activity in *A. fumigatus*. In addition, trehalose assays of *tslA* and *tslB* null mutants suggest that while both TslA and TslB are involved in trehalose biosynthesis, these two proteins are not redundant and have multiple functions that remain to be fully elucidated in *A. fumigatus*.

The major phenotype associated with loss of TslA is a significant alteration in cell wall integrity as evidenced by cell wall stress assays. In addition, TEM and cell wall chitin and beta-glucan exposure assays strongly suggest that loss of TslA impacts cell wall homeostasis. To further understand the underlying mechanisms, we used an affinity purification approach followed by liquid chromatography-tandem mass spectrometry (LC-MS/MS) analysis of coprecipitating proteins with TslA. In *S. cerevisiae*, *Sc*Tsl1p and Tps3p interact with proteins that regulate cell wall rigidity and cell wall components in the spores, i.e., Pmt6p, an O-mannosyltransferase, and Sps2p (a protein expressed during sporulation), but there are no reports of interactions with cell wall biosynthesis enzymes (38). Surprisingly, however, we discovered that *A. fumigatus* TslA interacts with a class V chitin synthase, CsmA. To our knowledge, this is the first report of a protein-protein interaction between trehalose and cell wall biosynthesis proteins in fungi. However, *Sc*Tsl1p and Tps3p also interact with other enzymes in glycolysis, including other mitochondrial proteins. In our experiments, we also observed that TslA in *A. fumigatus* also interacts with enzymes in glycolysis, the pentose phosphate pathway, and mitochondrial proteins though these interactions remain to be validated.

Consequently, our data suggest that TslA plays a complex role in fungal carbon metabolism, cell wall homeostasis, and fungus-host interactions. For chitin production, G6P is converted into fructose 6-phosphate and then N-acetyl glucosamine, while UDP-glucose is the key building block to generate β-glucan. These substrates, G6P and UDP-glucose, are also the critical building blocks for trehalose biosynthesis. Perturbations in trehalose biosynthesis that occur when key proteins are lost through genetic mutation or in response to specific environments thus result in significant alterations in fungal carbon metabolism that may alter biosynthetic processes in the cell that require sugar-phosphate intermediates. Nevertheless, from the mRNA expression of the transcription factors in the cell wall integrity pathway (RlmA), the high-osmolarity glycerol–mitogen-activated protein kinase (HOG-MAPK) pathway (AtfA), the β-glucan synthase enzyme (FksA), and the class V chitin synthase enzyme (CsmA), we observed that the loss of TslA has no intrinsic effect on mRNA levels of these genes. These results suggest that the observed defects in cell wall homeostasis may be the result of the direct regulation of CsmA or other cell wall biosynthesis components by TslA.

*A. fumigatus* contains eight chitin synthase enzymes divided into seven classes (39, 40). However, only class V and class VII enzymes have an N-terminal myosin motor-like domain (MMD) (41–43). Fungal cells pack these enzymes into 60-nm-diameter microvesicles, called chitosomes, and transport them to the hyphal tip (44). Chitosomes merge with the apical cell membrane, and chitin synthase enzymes (Chs) are transported into the interior side of the cell membrane (45). However, MMD-Chs are also able to transport themselves along actin filaments to the fungal tip (34). In *Ustilago maydis*, chitosomes are not required for the cytoplasmic motility of class V chitin synthases (46, 47). MMD-Chs are usually found at the hyphal tip and septa, so they are proposed to be involved in polarized cell wall biosynthesis and septal formation (48). *A. fumigatus* possesses two chitin synthases with an MMD, called CsmA and CsmB (49). A *csmA* null mutant shows less chitin content in the conidial cell wall (49). Recently, Muszkieta et al. observed that CsmA is important for cell wall homeostasis (50). Loss of *csmA*, *csmB*, *chsF*, and *chsD* causes formation of a disorganized cell wall structure and significantly attenuates virulence *in vivo* (50).

In contrast, our results suggest that loss of TslA results in altered CsmA localization and an increase in chitin synthase activity. It is unknown how TslA binds to CsmA and affects its localization and activity. Localization of chitin synthase enzymes is essential for function, and their localization is dependent upon multiple regulatory steps, including posttranslational modifications, e.g., phosphorylation and dephosphorylation. For example, in *S. cerevisiae*, *Sc*Chs3 is phosphorylated by *Sc*Pkc1 under conditions of heat stress (51). *Sc*Sac1 phosphatase inhibits *Sc*Chs3 forward transportation, while *Sc*Pik1 overexpression promotes forward movement (52). Both *Sc*Sac1 and *Sc*Pik1 are important for Golgi trafficking to the plasma membrane (52). Furthermore, phosphorylation and dephosphorylation of *Sc*Chs3 are necessary for guiding Chs3 to the septum in each cell cycle stage (53). Lenardon et al. showed that *C. albicans* Chs3 (*Ca*Chs3), a major enzyme for chitin synthesis, is phosphorylated at Ser139 in *C. albicans* (54). Mutations at the site revealed that both phosphorylation and dephosphorylation of *Ca*Chs3 are crucial for the localization and function of *Ca*Chs3, including the polarized growth. However, kinases regulating phosphorylation of *Ca*Chs3 are still unknown (54). Consequently, it is possible that the mechanism behind altered CsmA localization in the absence of TslA is related to alteration of CsmA phosphorylation. In addition to the phosphorylation, as mentioned above, chitin synthase localization is also associated with actin filaments (34). Therefore, it is possible that TslA may stabilize the chitin synthase and actin complex to help direct localization and activity. We found from the LC-MS/MS data that TslA did not pull down ActA but did pull down an actin cytoskeleton protein (VIP1) and an actin-bundling protein (Sac6). Moreover, we observed that TslA localized in the cytosol along the hyphae without any obvious specific TslA localization sites, i.e., hyphal tips (Fig. 8D) (see Fig. S1 in the supplemental material). Furthermore, in filamentous fungi, microtubule-based intracellular trafficking plays an important role in the dynamics of various vesicles and proteins (55, 56). It is possible that CsmA localization is involved with both actin filaments and the microtubule-based mechanism (34). Additional research is needed to define the molecular mechanism through which TslA regulates CsmA localization and/or activity.

Importantly, the fungal cell wall is not only important for fungal survival but also essential for interactions with the host immune system (57). The balance between the host immune response and virulence of the fungi is an important factor that determines the fate of both fungal pathogens and hosts (57). Chitin plays an important role in the immune response to fungi. For example, chitin has an immunomodulatory effect on the host by shifting the immune response from a T_H_1 response to a more T_H_2-like response that can have an impact on fungal survival inside the host (58). Here, we observed increased levels of chitin exposure and content in the cell wall for *A. fumigatus* in the absence of TslA. We also noted the presence of electron-dense material on the exterior of Δ*tslA* hyphae. This electron-dense material could be galactosaminogalactan (GAG), a major component of the *A. fumigatus* extracellular matrix (59). GAG is an adhesin that is essential for biofilm formation (59, 60). GAG also has immune-modulatory effects inducing T_H_2 lineage proliferation (59). Furthermore, exogenous GAG inhibits human proinflammatory cytokine production through interleukin-1 (IL-1) signaling by inducing IL-1 receptor antagonist (61). However, it remains unclear how various cell wall compositions impact the inflammatory response and disease outcomes in IPA murine models. Therefore, additional studies are needed to investigate the connections among loss of TslA, cell wall components, and the observed altered host immune response. One translationally relevant future research direction is to examine the effects of TslA loss on the efficacy of a chitin synthase inhibitor such as nikkomycin Z. As TslA and other trehalose biosynthesis proteins have a profound effect on fungal cell wall homeostasis, further investigation into these molecular mechanisms may reveal novel targets or approaches for therapeutic development.

In conclusion, our results suggest that both TslA and TslB are involved in the biosynthesis of trehalose in *A. fumigatus*. However, the mechanisms behind the regulation of trehalose production by these two proteins are still unclear. What is clear is that TslA has an unexpected additional so-called moonlighting role in regulating chitin synthase activity. On the basis of the impact of TslA loss on CsmA localization, we speculate that TslA might be critical for the proper localization of key trehalose biosynthesis proteins such as OrlA. Importantly, these results strongly suggest that trehalose-related proteins are important for cell wall biosynthesis not only for their role in carbon metabolism regulation but also from direct physical interactions with cell wall biosynthesis enzymes. A more fundamental understanding of the underlying mechanisms linking trehalose and cell wall biosynthesis may uncover potential novel antifungal targets and will enhance our understanding of *A. fumigatus*-host interactions.

## MATERIALS AND METHODS

### Fungal strains, media, and growth conditions.

*Aspergillus fumigatus* strain CEA17 (a uracil auxotroph strain lacking a *pyrG* gene) was used to generate *tslA*, *tslB*, and *tslA*/*B* null mutants (62). A *ku80* strain (a uracil auxotroph strain lacking *pyrG* and *akuB* genes) was used to generate S-tagged and Flag-tagged strains for pulldown and coimmunoprecipitation experiments (62, 63). Glucose minimal media (GMM) containing 1% glucose were used to grow the mutants along with a wild-type strain, CEA10 (CBS144.89), at 37°C in 5% CO_2_ if not stated otherwise (64). The conidia from each strain were collected by the use of 0.01% Tween 80 after 72 h of incubation at 37°C in 5% CO_2_. Fresh conidia were used in all experiments.

### Strain construction and fungal transformation.

Gene replacements and reconstituted strains were generated as previously described (15, 35). All strains are listed in Table S1 in the supplemental material. PCR and Southern blotting were used to confirm the mutant strains (15). Real-time reverse transcriptase PCR was used to confirm expression of the reintroduced gene (65). To generate the single-null mutant, *A. parasiticus pyrG* from pJW24 was used as a selectable marker (20). To generate a double-null mutant strain and reconstituted strains of single-null mutants, we utilized a *ptrA* marker, which is a pyrithiamine resistance gene from *A. oryzae* (21). To generate reconstituted strains of the double-null mutant, we utilized *hygB*, which is a hygromycin B phosphotransferase gene, as a hygromycin resistance marker (22). For S-tagged strains, an S-tag coding sequence was introduced along with *A. fumigatus pyrG* (*AfpyrG*) into the C terminus of proteins of interest, i.e., TslA and TslB (30, 31). For coimmunoprecipitation experiments, we introduced a Flag tag together with *ptrA* as a marker into the C terminus at the loci encoding proteins of interest, e.g., CsmA, in the TslA-S tag background (66). In localization experiments, we generated C-terminal GFP-tagged CsmA in both the wild-type (CEA17) and Δ*tslA* strain backgrounds by using *pyrG* and *ptrA* as selectable markers, respectively. After the constructs were generated, polyethylene glycol-mediated transformation of fungal protoplasts was performed as previously described (67). For the *ptrA marker* transformation, we added pyrithiamine hydrobromide (Sigma; catalog no. P0256) to 1.2 M sorbitol media (sorbitol minimal media [SMM]) at 0.1 mg/liter (21). For the *hygB marker* transformation, we recovered the strains containing the *hygB* marker by adding hygromycin B (Calbiochem; catalog no. 400052) into the 0.7% SMM agar overlay at 150 μg/ml the day after transformation (22).

10.1128/mBio.00056-17.2 TABLE S1 Strains in this study. Download TABLE S1, DOCX file, 0.1 MB. Copyright © 2017 Thammahong et al. 2017 Thammahong et al. This content is distributed under the terms of the Creative Commons Attribution 4.0 International license.

10.1128/mBio.00056-17.3 TABLE S2 Protein identities of LC-MS/MS from TslA-S tag pulldown assays. Download TABLE S2, XLSX file, 0.4 MB. Copyright © 2017 Thammahong et al. 2017 Thammahong et al. This content is distributed under the terms of the Creative Commons Attribution 4.0 International license.

### Trehalose measurement.

Trehalose content in conidia and mycelia was measured as previously described (15). Briefly, a total of 2 × 10^8^ conidia were used for the conidial stage of the trehalose assay, and 1 × 10^8^ conidia were cultured overnight in 10 ml liquid glucose minimal medium (LGMM) for the mycelial stage as described by d’Enfert and Fontaine (1997) (68). Cell-free extracts were then tested for trehalose levels according to the glucose assay kit protocols (Sigma; catalog no. GAGO20). Results from biological triplicate experiments were averaged, standard deviation calculated, and statistical significance determined (*P* < 0.05) with an unpaired two-tailed Student’s *t* test.

### Cell wall-perturbing agents and antifungal agents.

Several cell wall-perturbing agents, namely, Congo red (CR) (Sigma catalog no. C6277), calcofluor white (CFW) (fluorescent brightener 28; Sigma catalog no. F3543), and caspofungin (CPG) (Cancidas; Merck & Co., Inc.), were utilized for cell wall integrity tests. CR, CFW, or CPG was added into GMM plates at a final concentration of 1 mg/ml, 50 μg/ml, or 1 μg/ml, respectively. Dropout assays were performed by plating serial dilutions of 1 × 10^5^ to 1 × 10^2^ conidia in a 5-μl drop of each strain. The plates were cultured at 37°C in 5% CO_2_, and the images were taken at 48 h. This experiment was performed in three biological replicates (15).

### Cell wall MAMP exposure.

Calcofluor white (CFW), fluorescein-labeled wheat germ agglutinin (WGA) (Vector Laboratories; catalog no. FL-1021), and soluble dectin-1 staining was performed as previously described (58, 69). Briefly, each fungal strain was cultured until it reached the germination stage on liquid glucose minimal media. The hyphae were UV irradiated at 6,000 mJ/cm^2^. The micrographs were taken using the Z-stack of the fluorescence microscope, a Zeiss HAL 100 microscope (Carl Zeiss Microscopy LLC, Thornwood, NY), equipped with a Zeiss AxioCam MRm camera. The intensity was analyzed using ImageJ, and the corrected total cell fluorescence (CTCF) was calculated (69, 70). Data are presented as means ± standard errors (SE) corresponding to 15 images from three biological replicates.

### Transmission electron microscopy.

The cell walls of the wild-type strain (CEA10) and the Δ*tslA* and *ΔtslA+tslA* strains were examined by using TEM as previously described (19, 39). All TEM images were taken at 100 kV on a JEOL TEM 1010 microscope (JEOL, Tokyo, Japan) equipped with a digital camera (XR-41B; Advanced Microscopy Techniques). Cell wall thickness was analyzed using ImageJ (69). Data are presented as means ± SE of 10 measurements from two biological replicates of each strain.

### Proteomic assay, pulldown assay, and coimmunoprecipitation.

In the pulldown assays for the S tag, 10^8^ conidia of the wild-type and S-tagged strains were incubated in 100 ml liquid GMM medium at 30°C for 8 h and switched to 37°C for 16 h (250 rpm). The mycelia from each strain were collected and lyophilized overnight. Proteins were extracted as previously described (30). Sample supernatants were measured to estimate protein concentrations using the Bradford method (Bio-Rad, Hercules, CA). For the purification step, 300 μl of S protein agarose slurry (Novagen) (150-μl packed bead volume) was added per 100 mg of protein and incubated at 4°C using rotary agitation for 1 h and previously described purification steps (30). The supernatant was loaded into 10% mini-protein precast gels (Bio-Rad). The gel was stained with Bio-Safe Coomassie blue (Bio-Rad) for 3 h. The bands were cut and submitted for mass spectrometry analysis (LC-MS/MS) at The Vermont Genetics Network, University of Vermont, Burlington, VT.

### Co-IP with *S*-protein beads and anti-Flag magnetic beads.

To perform coimmunoprecipitation assays, C-terminal Flag-tagged CsmA strains were generated in the S-tagged TslA background. S-protein bead Co-IP experiments were performed in the same way as the previously described S-protein bead pulldown experiments. To perform reciprocal coimmunoprecipitation assays, C-terminal Flag-tagged CsmA strains were used. An IP buffer was used followed by affinity purifications with anti-Flag M2 magnetic beads (Sigma) as previously described (66). Proteins were transferred from a 10% SDS-PAGE gel onto a polyvinylidene difluoride (PVDF) membrane for a Western blot assay using a Trans-Blot turbo transfer system (Bio-Rad). S-tagged TslA was detected using a rabbit anti-S-tag antibody (ICL) at 1:5,000 dilution and a goat anti-rabbit IgG (H+L) horseradish peroxidase (HRP) antibody (Thermo Scientific) at 1:10,000 dilution. For the Flag-tagged CsmA, a mouse monoclonal anti-Flag M2 antibody (F1804; Sigma) was used at 1:10,000 dilution as a primary antibody followed by an anti-mouse IgG HRP conjugate (W4021; Promega) used at 1:2,500 dilution as a secondary antibody. Chemiluminescence detection was performed using a Clarity Western ECL substrate (Bio-Rad) and a FluorChem FC2 imager (Alpha Innotech). For loading controls, an anti-tubulin antibody (Sigma; catalog no. T5192) (human) was utilized.

### Chitin synthase activity assay.

A total of 10^8^ conidia of each fungal strain were grown at 37°C for 24 h in 10 ml of liquid GMM at 250 rpm. The mycelia were collected for preparation of membrane fractions by centrifugation at 100,000 × *g* for 40 min at 4°C as described before. After that, the nonradioactive chitin synthase activity assay was performed in a 96-well plate as previously described (32, 33).

### Murine model of invasive pulmonary aspergillosis.

CD1 female mice (6 to 8 weeks old) were used in chemotherapeutic murine model experiments as previously described (35). Mice were obtained from Charles River Laboratories, Inc. (Raleigh, NC). For survival studies and histopathology, 10 mice per *A. fumigatus* strain (including strains CEA10, Δ*tslA*, and *ΔtslA+tslA*) were inoculated intranasally with 1 × 10^6^ conidia in 40 μl of phosphate-buffered saline (PBS) and monitored three times a day. Mice were observed for 14 days after the *A. fumigatus* challenge. Any animals showing distress were immediately humanely sacrificed and recorded as deaths within 24 h. No mock-infected animals perished in any of the experiments. Statistical comparison of the associated Kaplan-Meier curves was conducted with log rank tests (71). Lungs were removed from all mice sacrificed at different time points during the experiment for fungal burden assessment and histopathology.

### Histopathology.

The chemotherapeutic murine model was performed additionally for histopathology. Three mice in each group (including the CEA10, Δ*tslA*, and *ΔtslA+tslA* strain groups) were humanely euthanized at day 2 and day 4 postinoculation. Lungs were harvested from each group and fixed in 10% formalin before embedding in paraffin was performed. Sections (5 μm in thickness) were taken and stained with either H&E (hematoxylin and eosin) or GMS (Gomori’s methenamine silver stain) as previously described (72). Slides were analyzed microscopically with a Zeiss Axioplan 2 imaging microscope (Carl Zeiss Microimaging, Inc., Thornwood, NY) fitted with a QImaging Retiga-SRV Fast 1394 red-green-blue (RGB) camera. The analysis was performed in Phylum Live 4 imaging software. Images were captured at ×50 magnification as indicated in each image.

### *In vivo* fungal burden.

Quantitative analysis of fungal growth in infected mouse lungs was performed after lungs were harvested at day 4 postinoculation with a quantitative PCR as previously described (36). Values were averaged for the CEA10, Δ*tslA*, and Δ*tslA+tslA* strains at each time point and compared using the Mann-Whitney-corrected *t* test.

### Collection and analysis of bronchoalveolar lavage fluid (BALF).

At the indicated time after *A. fumigatus* instillation, mice were euthanized using CO_2_. Bronchoalveolar lavage fluid (BALF) was collected by washing the lungs with 2 ml of PBS containing 0.05 M EDTA. BALF was then centrifuged and the supernatant collected and stored at −20°C until analysis. BAL fluid cells were resuspended in 200 µl of PBS and counted on a hemocytometer to determine total cell counts. Cells were then spun onto glass slides using a Thermo Scientific Cytospin4 cytocentrifuge and were subsequently stained using a Diff-Quik staining kit (Electron Microscopy Sciences) for differential cell counting. Assays for analysis of cytokines and chemokines from BALF were performed by using a Luminex system as previously described (69).

### Ethics statement.

This study was carried out in strict accordance with the recommendations given in the *Guide for the Care and Use of Laboratory Animals* of the National Institutes of Health. The animal experimental protocol was approved by the Institutional Animal Care and Use Committee (IACUC) at Dartmouth College (protocol number cram.ra.1).

### Statistical analysis.

All statistical analyses were done with Prism 5 software (GraphPad Software, Inc., San Diego, CA). All error bars represent standard errors of the means.
