# Supplementary figures and images for: Aspergillus fumigatus Trehalose-Regulatory Subunit Homolog Moonlights To Mediate Cell Wall Homeostasis through Modulation of Chitin Synthase Activity
Source: mBio. 2017 Apr 25;8(2):e00056-17. doi: 10.1128/mBio.00056-17 (PMC5405227; doi:10.1128/mBio.00056-17)

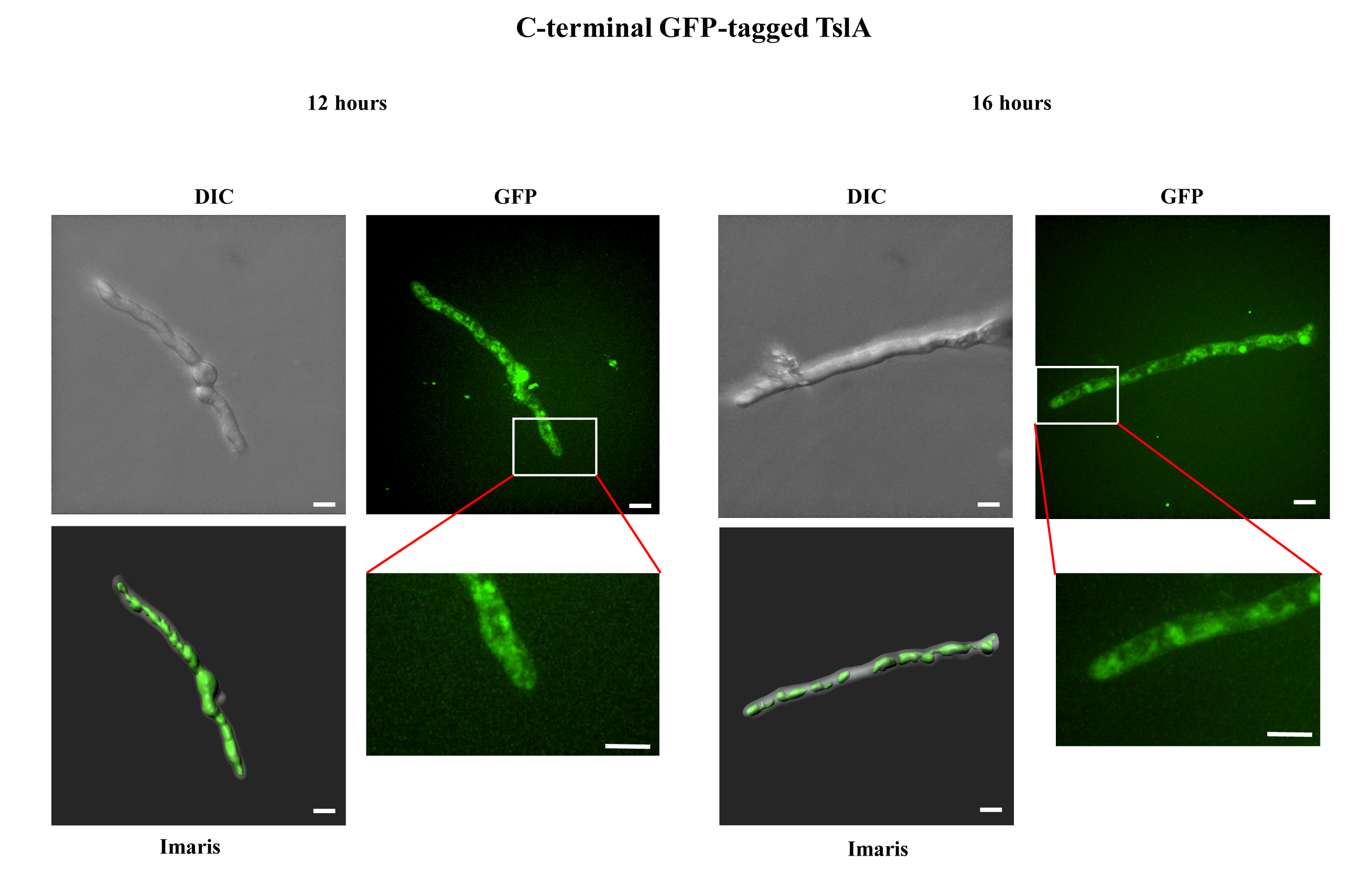

Supplement: FIG S1 [file mbo002173290sf1.tif]
