# Supplementary material for: Aspergillus fumigatus Trehalose-Regulatory Subunit Homolog Moonlights To Mediate Cell Wall Homeostasis through Modulation of Chitin Synthase Activity
Source: mBio. 2017 Apr 25;8(2):e00056-17. doi: 10.1128/mBio.00056-17 (PMC5405227; doi:10.1128/mBio.00056-17)
Supplement: TABLE S1 [file mbo002173290st1.docx]

| Strains | Genotype | References |
| --- | --- | --- |
| Fungal strains | | |
| CEA17 | CEA10 *pyrG^-^* | (1) |
| *Ku80* | CEA10 *pyrG^-^ akuB^-^* | (2) |
| Δ*tslA* (AFUB_089470) | CEA17 *tslA::pyrG* | This study |
| Δ*tslB* (AFUB_021090) | CEA17 *tslB::pyrG* | This study |
| Δ*tslA/B* | CEA17 *tslA::pyrG, tslB::ptrA* | This study |
| Δ*tslA+TslA* | CEA17 *tslA::pyrG, tslA:ptrA* | This study |
| Δ*tslB+TslB* | CEA17 *tslB::pyrG, tslB:ptrA* | This study |
| Δ*tslA/B+TslA* | CEA17 *tslA::pyrG, tslB::ptrA, tslA:hygB* | This study |
| Δ*tslA/B+TslB* | CEA17 *tslA::pyrG, tslB::ptrA, tslB:hygB* | This study |
| TslA-S tag | *Ku80 tslA:S tag:pyrG* | This study |
| TslB-S tag | *Ku80 tslB:S tag:pyrG* | This study |
| TslA-S tag; CsmA-Flag | *Ku80 tslA:S tag:pyrG, CsmA:Flag tag:ptrA* | This study |
| CsmA-Flag | *Ku80 CsmA:Flag tag:ptrA* | This study |
| CsmA-GFP | CEA17 *csmA:GFP:pyrG* | This study |
| Δ*tslA*; CsmA-GFP | CEA17 *tslA::pyrG, csmA:GFP:hygB* | This study |
| Δ*tslA*; CsmA-GFP+*TslA* | CEA17 *tslA::pyrG, csmA:GFP:hygB, TslA:ptrA* | This study |
| TslA-GFP | CEA17 *tslA::GFP::pyrG* | This study |
| Plasmids | | |
| pJW24 | *A. parasiticus pyrG* | Gift from Keller lab |
| pSD51.1 | *A. oryzae ptrA* | Gift from Calvo lab |
| pBC-hygro | Hygromycin B resistance gene | FGSC (Silar, FGN 42:73) |
| pAO81 | *S-tag:pyrG* | FGSC (Yang L, *et al.* 2004) |
| p3xFLAG-CMV 10 | *3xFlag* | Gift from Kettenbach lab |
| pSD79.1 | *3xFlag:pyrG* | This study |
| pSD89.1 | 3xFlag:ptrA | This study |
| pFNO3 | GFP:*AfpyrG* | FGSC (Euk Cell 3:1359-62) |
| pTH1067.9 | GFP:*hygB* | FGSC (Hammond *et al*, 2011) |

**References**

1. d'Enfert C. 1996. Selection of multiple disruption events in *Aspergillus fumigatus* using the orotidine-5'-decarboxylase gene, *pyrG*, as a unique transformation marker. Curr Genet 30:76-82.

2. da Silva Ferreira ME, Kress MR, Savoldi M, Goldman MH, Hartl A, Heinekamp T, Brakhage AA, Goldman GH. 2006. The *akuB*(*KU80*) mutant deficient for nonhomologous end joining is a powerful tool for analyzing pathogenicity in *Aspergillus fumigatus*. Eukaryot Cell 5:207-11.
